# Supplementary figures and images for: Salmonella enterica serovar Typhimurium chitinases modulate the intestinal glycome and promote small intestinal invasion
Source: PLoS Pathog. 2022 Apr 28;18(4):e1010167. doi: 10.1371/journal.ppat.1010167 (PMC9049507; doi:10.1371/journal.ppat.1010167)

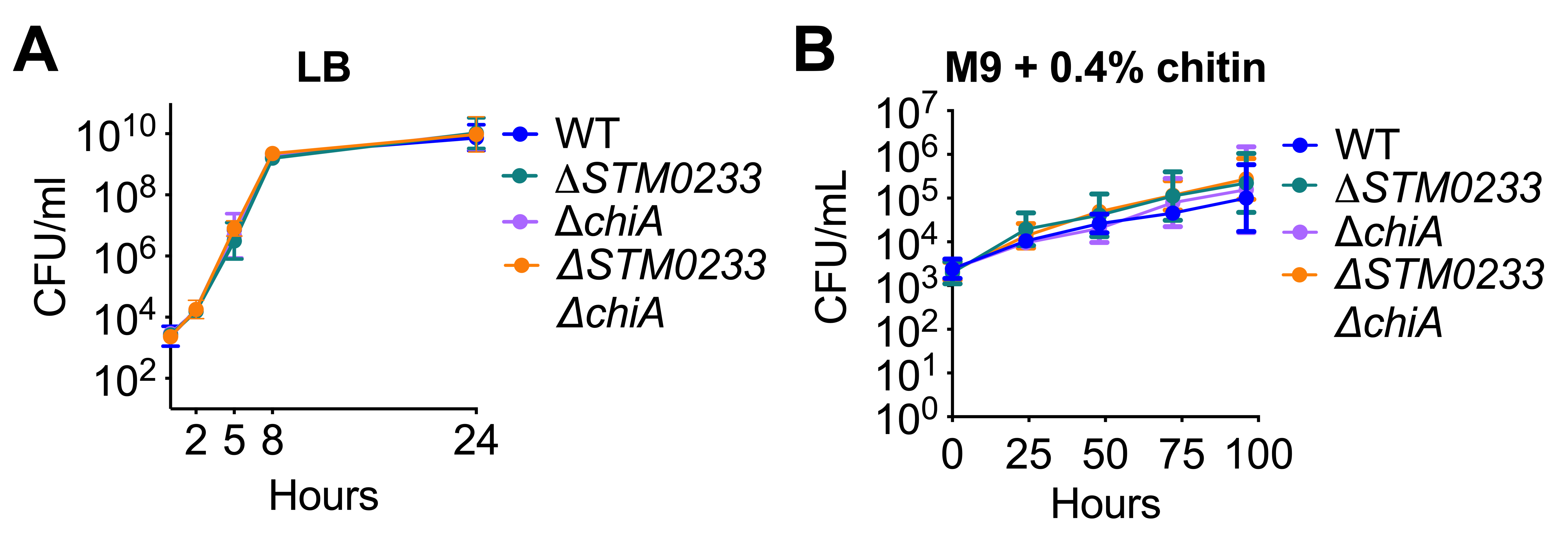

Supplement: S1 Fig — (A) Growth of S. Typhimurium strains in LB broth at 37°C. WT n = 5, chitinase-deficient strains n = 3. Points represent geometric mean ± geometric SD. (B) Growth of S. Typhimurium strains in M9 minimal medium +0.4% colloidal chitin at 37°C. n = 3. Points represent geometric mean ± geometric SD. Statistics: Mixed-effect analysis and Tukey’s multiple comparisons test. (TIFF) [file ppat.1010167.s001.tiff]

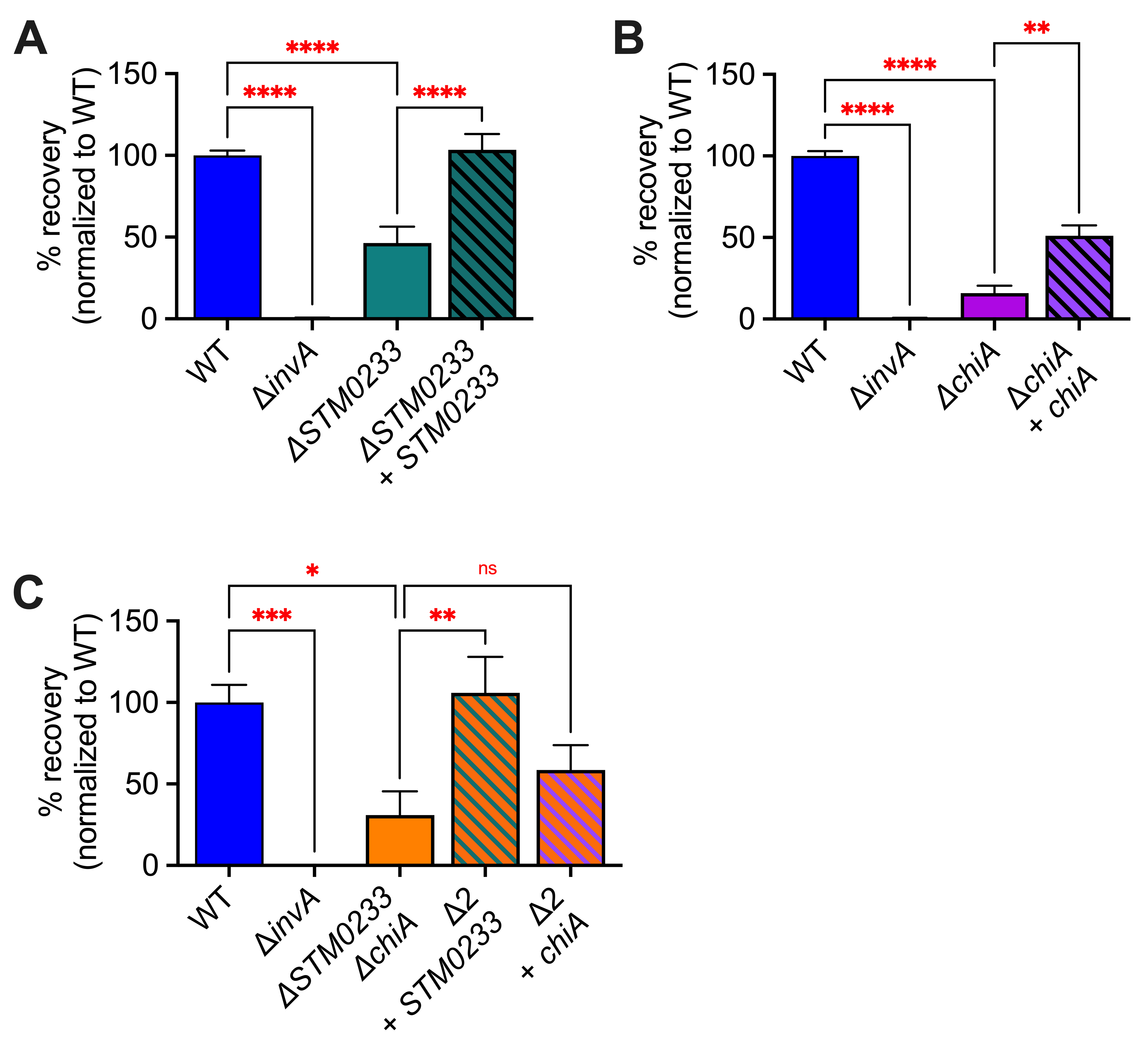

Supplement: S2 Fig — Chitinase genes were inserted into the Tn7 locus of chitinase-deficient S. Typhimurium. Gentamicin protection assay of S. Typhimurium infected (MOI:1) small intestinal epithelial cells (IPEC-1). (A) Invasion of ΔchiA and chiA complemented strain. n = 8. (B) Invasion of ΔSTM0233 and STM0233 complemented strain. n = 8. (C) Invasion of ΔSTM0233 ΔchiA (Δ2) and Δ2 strain complemented with chiA or STM0233. n = 6. Percent recovery of each strain was normalized to WT recovery. Bars represent mean ± SEM. Statistics: One-way ANOVA with Dunnett’s multiple comparison test. (TIFF) [file ppat.1010167.s002.tiff]

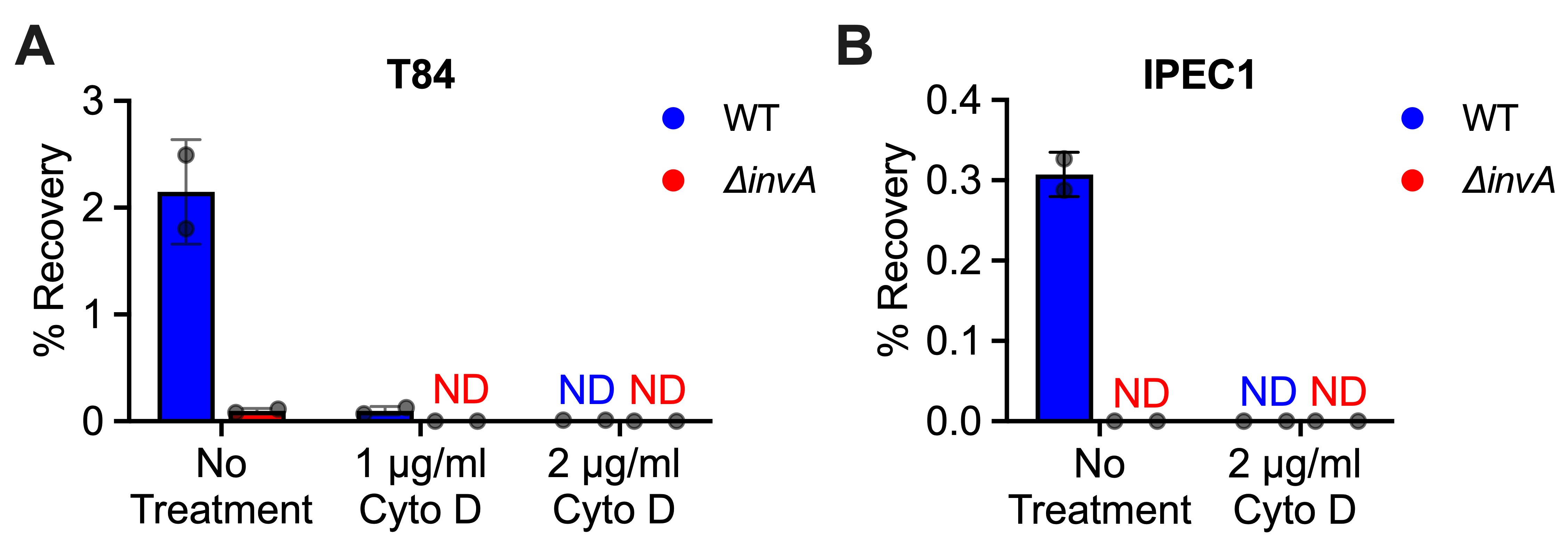

Supplement: S3 Fig — (A) Gentamicin protection assay of S. Typhimurium infected (MOI:1) colonic epithelial cells (T84) after treatment of epithelial cells with cytochalasin D (1–2 μg/mL). (B) Gentamicin protection assay of S. Typhimurium infected (MOI:1) small intestinal epithelial cells (IPEC-1) after treatment of epithelial cells with cytochalasin D (2 μg/mL). n = 2. Bars represent mean ± SD. (TIFF) [file ppat.1010167.s003.tiff]

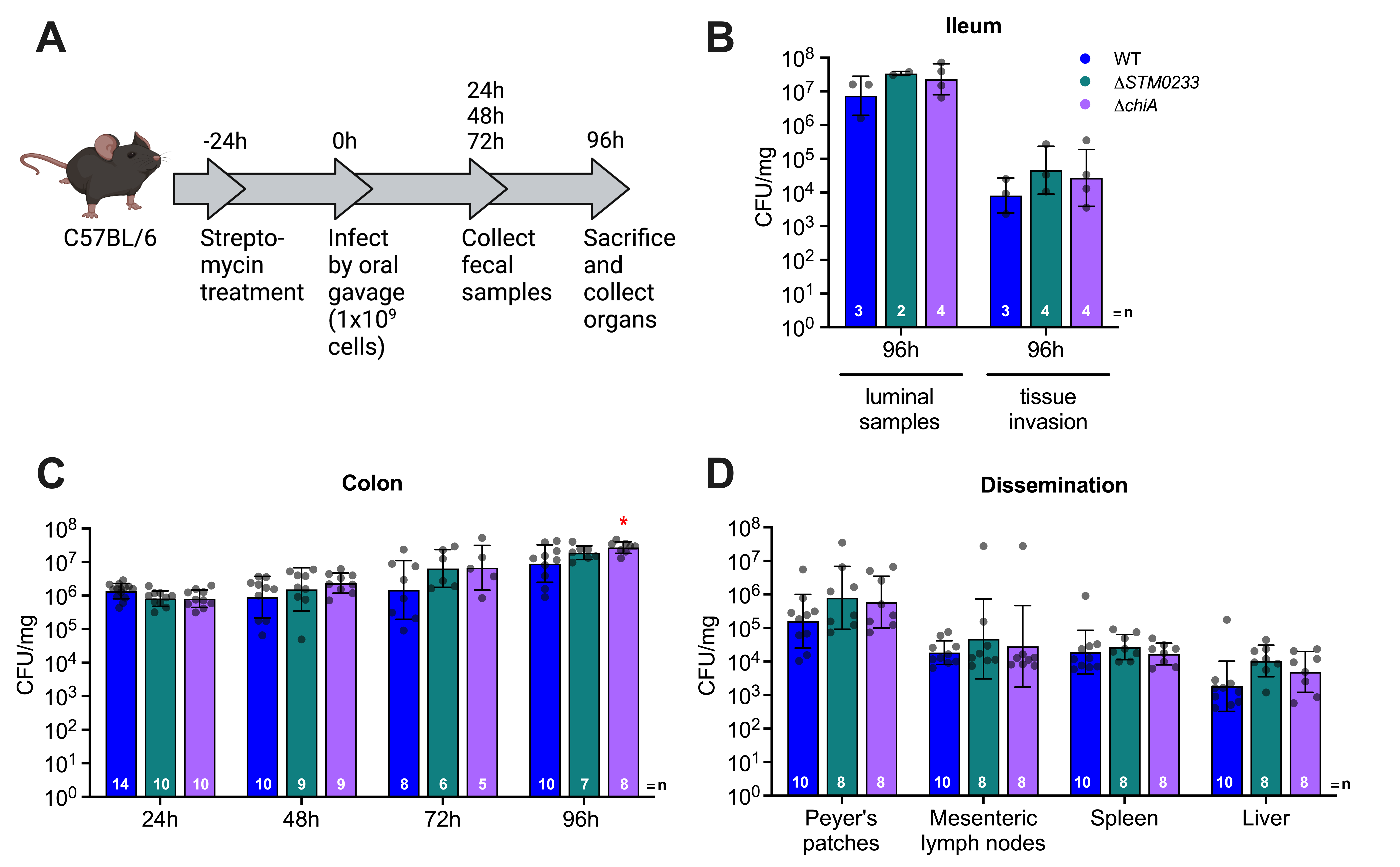

Supplement: S4 Fig — (A) Streptomycin pre-treatment mouse model of 96 h S. Typhimurium infection. (B) Luminal samples from the terminal ileum were collected at 96 hpi to determine S. Typhimurium colonization. Invasion was determined with a gentamicin protection assay performed on the terminal ileum. WT n = 3, ΔSTM0233 n = 3, ΔchiA n = 4. (C) S. Typhimurium colonies recovered from fecal samples collected at 24, 48, and 72 hpi. Fecal samples at 96 hpi were collected directly from the lumen of the colon. Invasion of colonic tissue was determined with a gentamicin protection assay. WT n = 10, ΔSTM0233 n = 8, ΔchiA n = 8. (D) Colonization of the Peyer’s patches, mesenteric lymph nodes, spleen, and liver after 96 h of intragastric infection. WT n = 10, ΔSTM0233 n = 8, ΔchiA n = 8. Bars represent geometric mean ± geometric SD. Statistics: (B-D) Stars indicate significance compared to the WT control by one-way ANOVA with Dunnett’s multiple comparison test. * = p<0.05. (TIFF) [file ppat.1010167.s004.tiff]

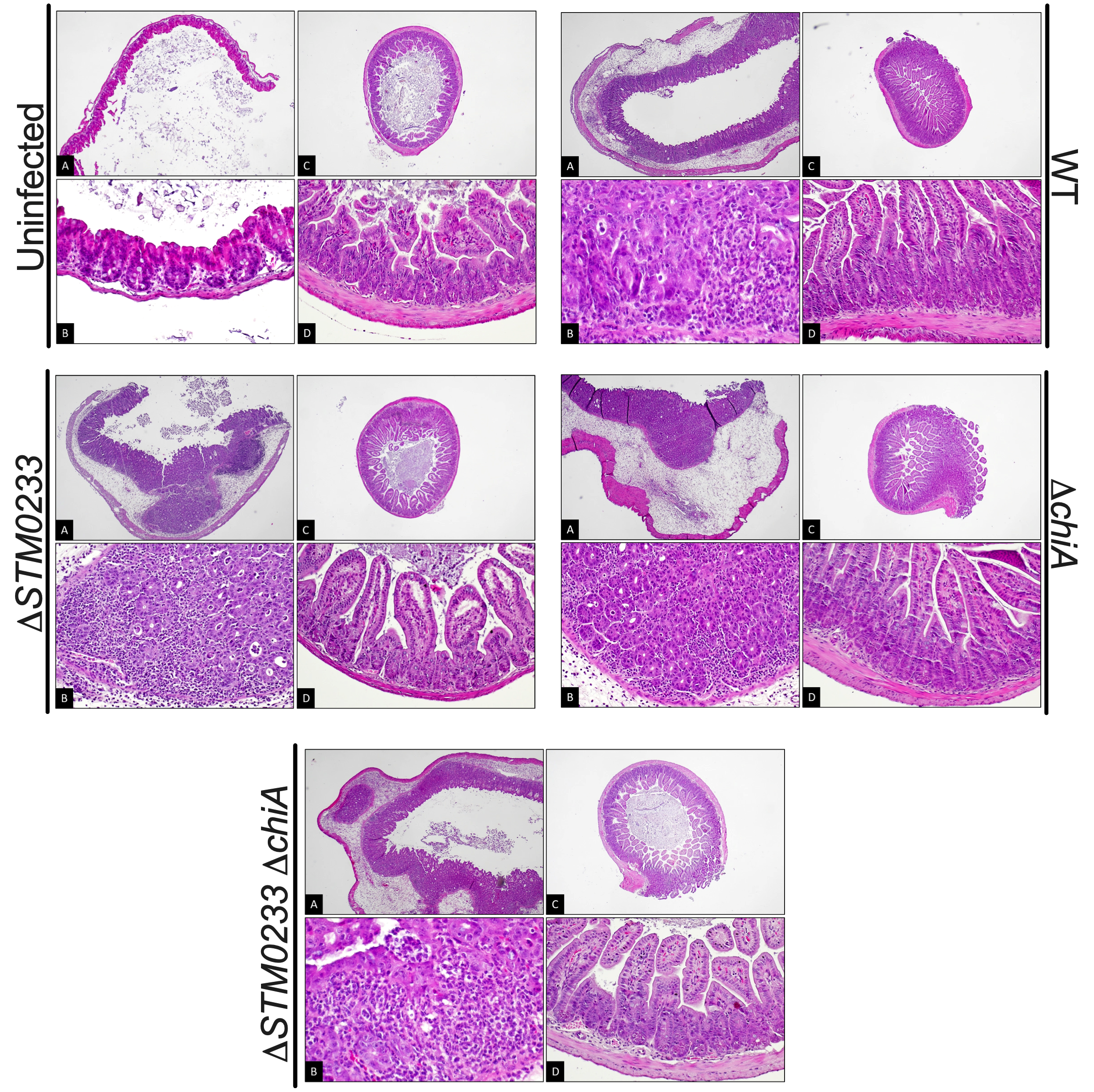

Supplement: S5 Fig — Uninfected: (A) & (B) Cecum–intestine intact without any signs of inflammation [A: low power, hematoxylin eosin, original magnification 40x; B: intermediate power, hematoxylin-eosin, original magnification 200x]. (C) & (D) Ileum–intestine intact without any signs of inflammation [C: low power, hematoxylin eosin, original magnification 40x; D: intermediate power, hematoxylin eosin, original magnification 200x]. WT: (A) & (B) Cecum–moderate inflammation (moderate submucosal edema with lamina propria neutrophilic infiltration with cryptitis, crypt abscess and decrease in goblet cells) [A: low power, hematoxylin-eosin, original magnification 40x; B: high power, hematoxylin-eosin, original magnification 400x]. (C) & (D) Ileum–intestine intact without any signs of inflammation [C: low power, hematoxylin-eosin, original magnification 40x; D: intermediate power, hematoxylin-eosin, original magnification 200x]. ΔSTM0233: (A) & (B) Cecum–moderate inflammation (moderate submucosal edema with lamina propria neutrophilic infiltration with cryptitis, crypt abscess and decrease in goblet cells) [A: low power, hematoxylin-eosin, original magnification 40x; B: intermediate power, hematoxylin-eosin, original magnification 200x]. (C) & (D) Ileum–intestine intact without any signs of inflammation [C: low power, hematoxylin-eosin, original magnification 40x; D: intermediate power, hematoxylin-eosin, original magnification 200x]. ΔchiA: (A) & (B) Cecum–moderate inflammation (profound submucosal edema with lamina propria neutrophilic infiltration with cryptitis, crypt abscess and decrease in goblet cells) [A: low power, hematoxylin-eosin, original magnification 40x; B: intermediate power, hematoxylin-eosin, original magnification 200x]. (C) & (D) Ileum–intestine intact without any signs of inflammation [C: low power, hematoxylin-eosin, original magnification 40x; D: intermediate power, hematoxylin-eosin, original magnification 200x]. ΔSTM0233 ΔchiA: (A) & (B) Cecum–moderate inflammation ( [file ppat.1010167.s005.tiff]

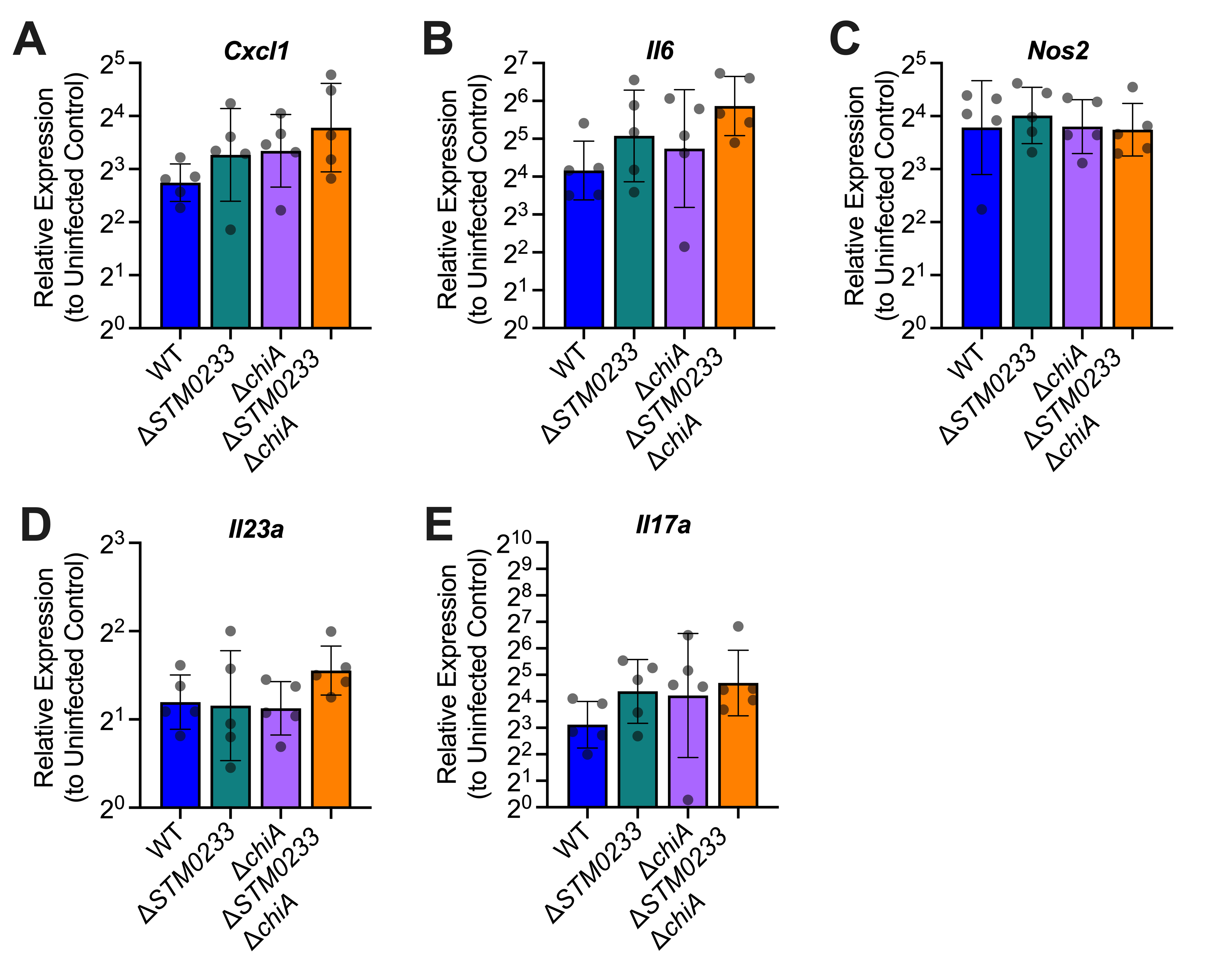

Supplement: S6 Fig — (A-E) mRNA expression of innate immune genes extracted from ileal tissue of S. Typhimurium infected mice 48 hpi. Expression is normalized to the housekeeping gene actb as well as gene expression in uninfected mice. n = 8 per group. Bars represent geometric mean ± geometric SD. There was no statistical significance (α = 0.05) comparing groups to the WT control by one-way ANOVA with Dunnett’s multiple comparison test. (TIFF) [file ppat.1010167.s006.tiff]

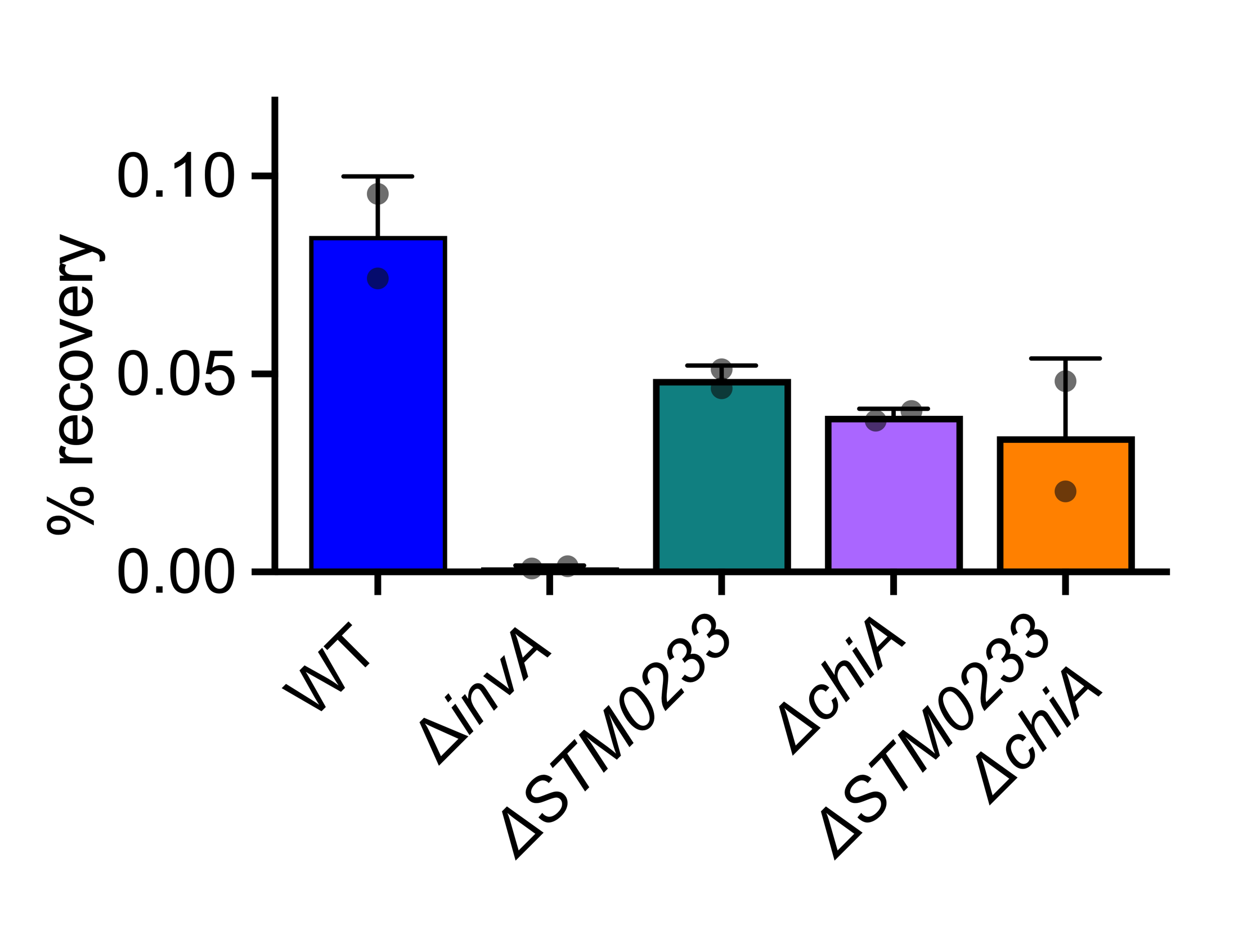

Supplement: S7 Fig — Gentamicin protection assay of S. Typhimurium infected IPEC-1 cells (MOI:1000) done concurrently with infection for the glycome analysis. n = 2. Bars represent mean ± SD. (TIFF) [file ppat.1010167.s007.tiff]

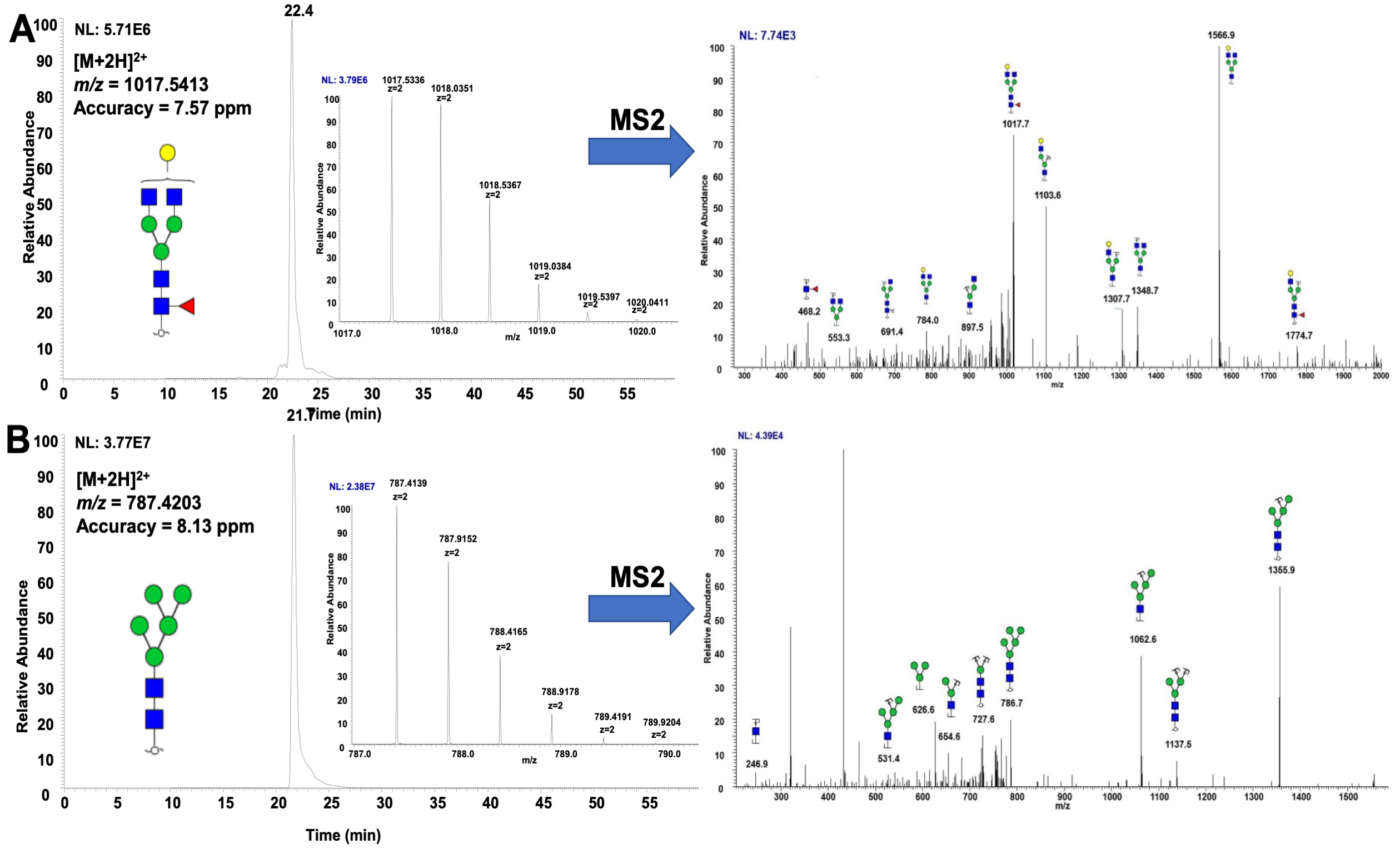

Supplement: S8 Fig — Examples of (A) complex and (B) high-mannose N-glycan identification through full MS and MS2. Insets are full MS spectra showing isotopic distribution envelops. The N-glycan compositions are identified through full MS within an m/z accuracy of 10 ppm. The putative N-glycan structures are further confirmed by matching the fragment ions detected in MS2 spectra. (TIFF) [file ppat.1010167.s008.tiff]
