# Supplementary material for: Salmonella enterica serovar Typhimurium chitinases modulate the intestinal glycome and promote small intestinal invasion
Source: PLoS Pathog. 2022 Apr 28;18(4):e1010167. doi: 10.1371/journal.ppat.1010167 (PMC9049507; doi:10.1371/journal.ppat.1010167)
Supplement: Attachment — Submitted filename: PPATHOGENS-D-21-02439R1 - Response to Reviewers.pdf [file ppat.1010167.s014.pdf]

RESPONSE TO REVIEWERS

Part I – Summary

<u>Reviewer #1:</u> In this study, Devlin et al investigate the role of chitinase-like glycoside hydrolases in the pathogenesis of Salmonella infection. Defined mutants lacking activity of two chitinase-like glycoside hydrolases, either as single mutants or in combination, exhibit decreased invasiveness and adhesion towards culture epithelial cells (IPEC-1 and T84 cell lines). Chitinase-deficient mutants are less invasive (about one order of magnitude) in the small intestine in a mouse model of Salmonella-induced colitis. ChiA is also required for efficient dissemination to systemic sites, but is dispensable for systemic replication. The defect of chitinase-deficient mutants in the mouse model is rescued by co-infection with the wild type, consistent with the fact that these chitinases are secreted. Evidence is presented that the glycome of cultured epithelial cells is changed upon Salmonella infection, and that this change is in part dependent on chitinase activity.

The main finding that Salmonella uses glycoside hydrolases to promote early invasion events in the small intestine is intriguing and novel, and should be of high interest to the audience of Plos Pathogens. The manuscript is well written. The majority of the conclusions are justified.

<u>Reviewer #2:</u> Manuscript by Devlin et al investigates the role of two annotated chitinases STM0018 (chiA) and STM0233 during Salmonella infection and pathogenesis. While ChiA was studied before, STM0233s role has not been investigated prior to this study. Authors demonstrate that both chitinases are upregulated in the mouse gut and facilitate epithelial cell adhesion and invasion. While wild type Salmonella that produces the two chitinases can hydrolyze the N-acetylglucosamine-containing glycans in the mouse gut and on epithelial cells, promoting the adhesion of the pathogen, mutants defective in one or both chitinases show reduced adhesion, invasion and dissemination to systemic organs. Finally the authors investigate the glycan profiles in wild type and the mutant bacteria to show how these chitinases increase the exposure of certain glycans including LewisX glycans so that the wild type Salmonella can increase its binding and facilitate virulence. Overall this is a well-performed study that increases of our understanding of initial steps of Salmonella pathogenesis and how this pathogen can bind and invade epithelial tissue. Study is supported by both in vitro and in vivo mouse experiments. There are few minor concerns.

<u>Reviewer #3:</u> This work relies on previous publications describing that chitin-degrading enzymes participate in bacterial pathogenesis. In this work, the authors address the role of two chitinases, in the pathogenesis of Salmonella Typhimurium. The work shows that chitinase mutants exhibit reduced adhesion to cultured intestinal epithelial cells, reduced invasion in the ileum and lower dissemination into systemic organs, compared to the wt strain. Chitinases also cause significant change into the glycan repertoire on intestinal epithelial cells, although some changes cannot be attributed to the glycolytic activity of the enzymes, and the underlying mechanism remains elusive. The manuscript is well-organizes and the writing is clear and focused. The topic is very important and timely, and holds a potentially significant contribution to the field of pathogenesis. The experiments are well-performed and the data is compelling. My main concern is that the molecular underpinnings are still very obscure and it remains unclear how does the enzymatic activity of chitinases executes all the observed phenotype. However, the findings in this work are novel and important, and will probably provide a solid base for further studies.

We thank the reviewers for their overall very favorable evaluation of our manuscript, highlighting that the study is well executed and that it addresses an important topic of high relevance to the

readers of PLOS Pathogens. We appreciate the detailed comments that helped us to further enhance the clarity of our manuscript. We addressed the reviewer's comments in detail below (line numbers are for marked up manuscript).

We also made some additional changes to the manuscript that should further enhance scientific accuracy and clarity.

1. Changed *Salmonella* to *S.* Typhimurium to follow PLOS guidelines
2. Added database accession numbers for genome and genes used in study
3. Fig. 3B: Added 24 hpi time point for intraperitoneal infection. We performed the 24 hpi experiment after submission to exclude the possibility of an early colonization defect. 24h after intraperitoneal infection, colonization levels in liver and spleen were equivalent to 48hpi with orogastric administration. Also at 24 hpi, *Salmonella* WT and chitinase mutants colonized liver and spleen to the same extent. This experiment confirmed that chitinases are required for small intestinal invasion but not for systemic dissemination. We therefore decided to add this experiment to the manuscript.
4. Line 438-440: We recently became aware that the m/z ratios for lewis x glycans can also describe lewis A glycans. We therefore clarified that these two isomers could not be differentiated in our experiment.
5. Line 735-739: We added more information on how the glycome analysis was performed.

Part II – Major Issues: Key Experiments Required for Acceptance

Reviewer #1:

I only have one concern regarding the interpretation of the glycomics data and the conclusion that "STM0233 and ChiA enhance Salmonella adhesion to epithelial cells likely due to the exposure of mannose and increase in Lewis X binding residues." This conclusion is primarily based on the experiment shown in Fig. 6. In this experiment, the authors have shown that Salmonella induces changes in the surface glycome of intestinal epithelial cells, but I am not convinced that STM0233 and ChiA are involved. If the process is driven by invading Salmonella bacteria, then the changes induced by STM0233 and ChiA could simply be due to differences in invasion (Fig. S6). I don't think the entire glycomics survey should be repeated with an invasion-deficient strain or in the presence of cytochalasin D. However, one simple experiment would be to quantify one or two putative substrate on epithelial cells and rule out that invasion per se is a driver of the observed changes. At a minimum, this limitation could be discussed in the text.

We appreciate the reviewers' concerns. We were also initially concerned that different invasion rates might change the epithelial cell surface glycome. However, a previous study on the epithelial cell glycome significantly mitigates these concerns. This study showed that invasion-deficient (*invA*) and WT *Salmonella* induce similar changes in the surface glycome of colonic epithelial cells during infection (Park et al. 2016, Mol Cell Proteomics [26]), which indicates that *Salmonella*-induced glycome changes are not dependent on invasion. We are using a different cell line than the previous study, but do not think the response will be entirely different in epithelial cell lines that are both derived from the intestines. Nevertheless, we aimed to perform glycomic analysis after incubating different glycan substrates with wild type and chitinase deficient *Salmonella*

strains as suggested by the reviewer. However, we encountered low recovery, which did not allow interpretation of the experimental results. We chose not to repeat the experiment because we aim to purify the chitinases, which will allow direct substrate analysis and functional testing of chitinases to be featured in a future study. We now clearly address these limitations in the manuscript (Line 407-412).

Reviewer #2:

1- Throughout the study authors investigate the binding of Salmonella to ileum or colon using in vitro and in vivo studies. One would expect that reduced Salmonella adhesion, invasion and dissemination would be also reflected to reduced cytokine profiles in the tissues where the author see an effect of chitinases such as ileum. It is not clear why the authors they chose to use the cecal tissue to investigate the cytokine production. It is not suprising that they do not observe major changes in the cecal cytokine profiles at one time point except increased levels of il17a production in the animals infected by the chitinase mutants. It would be more informatory to study the cytokine profiles in the relevant ileal tissue or even peyers patches to show whether the chitinases effect on tissue invasion would reflect to the cytokine expression in these tissues.

We agree with the reviewer that ileal gene expression is more relevant in regard to our observed phenotype in the ileum than cecal gene expression. We therefore repeated the 48 hpi mouse experiment and collected the terminal ileum for RNA extraction. We examined the cytokine expression in ileal tissue for cytokines that are highly relevant for *Salmonella* infection and/or were regulated in cecal tissue. *Il23, Il6, Cxcl1, and Il17a* trended towards higher expression in mice infected with chitinase-deficient *Salmonella* strains, but this was not statistically significant (new Fig. S7). We observed at most 2-3 fold difference between wild type and the double chitinase deficient strain. Notably, *Nos2* expression did not change when mice were infected with WT or chitinase deficient strains (new Fig. S7). These findings are now reported in lines 255-259. The small difference in expression of some inflammatory genes also did not translate into differences in histopathology of the ileum after infection with chitinase deficient strains (Fig. S5).

2- Can the authors test the pef mutant in vitro to see whether pef fimbriae binds to the the exposed Lewis X glycans? This experiment would increase the impact of the paper if the authors can make that small but very important mechanistic link.

We thank the reviewer for this suggestion. We indeed plan on studying in a future manuscript if chitinase activity mediates fimbriae binding. We aim to explore this mechanistic link, which will require the generation of multiple mutants, e.g. *pef chiA STM0233*, and purified chitinases. With the *pef* mutant strain we acquired, we would only be able to phenocopy the invasion defect of the chitinase mutants without direct mechanistic link. While we are excited to explore this possibility further, addressing it adequately will unfortunately exceed the revision timeline and the scope of this current manuscript.

Reviewer #3:

None

Part III – Minor Issues:

Reviewer #1:

Line 128: As written, it seems somewhat contradictory to me that the authors seek to investigate the role of chitinase activity during “Salmonella infection” and then assay transcription under laboratory conditions – maybe this could be reworded.

We agree with the reviewer and reworded this statement (line 139).

Fig. 1D and E: I am somewhat surprised that the invA mutant is recovered in lower numbers than the wild type. Would one expect that both strains are recovered in equal numbers after the cytochalasin D treatment since they both should adhere in a similar manner? It might be useful for the reader if the authors could discuss this potential caveat in the text.

We initially shared the reviewers’ confusion about the observed *invA* adhesion phenotype. However, has been previously shown that *invA* mutants demonstrate lower adhesion because of the lack of a functional T3SS-1. We added more background information about how the T3SS-1 also drives the stable adhesion to epithelial cells to clarify this observation in the manuscript (line 177-180).

Fig. 4: The y-axis is labelled as “arbitrary units” – aren’t these fold changes over untreated control mice?

The reviewer is correct, and we changed the y-axis to reflect this more accurately.

Reviewer #2:

The double mutant is only referred to as Δ2 only in Fig 4A through out the manuscript. It would be better for consistency to change it to ΔSTM0233 ΔchiA.

We updated the x-axis labels for Fig. 4A.

If there is a more methodical way to show the glycan data to help the reader understand the different glycans, it would really help the manuscript. Not the clarity but it is difficult to digest this data for someone who is nor familiar with glycans. The little images by the graphs are a good idea though.

We added a 5-digit code to identify each glycan in Fig 6 and Table S4 and included a description of the code in the figure legend. This will better help readers identify the glycans that we refer to. We have also added a supporting figure (Fig. S8) and corresponding sentences in the Method section to explain the structural identification (line 734-739).

Reviewer #3:

1. Line 181 – Are the genes expression upregulated compared to expression in LB? If so, how does the expression of the normalizing gene change between LB an luminal content? I would

suggest presenting the data as total copy numbers of mRNA (cDNA) per ml/gr/cfu, which would be a more informative.

We reworded the statement to better reflect that expression is compared to LB (line 198). *Gmk* expression has been widely used as a housekeeping gene for various serovars of *Salmonella enterica*, including under *in vivo* conditions (Thiennimitr *et al.* 2011. Intestinal Inflammation Allows *Salmonella* to Use Ethanolamine to Compete with the Microbiota. *PNAS* 108 (42): 17480–85.) Expression of *gmk* was also shown to be stable under various growth conditions, which we added as a statement in the method section (line 688-689). Based on this, we believe that overall expression of *gmk* is representative of *Salmonella* abundance. As the samples needed to be immediately frozen for RNA extraction, we did not quantify the amount of *Salmonella* CFUs in the samples we used for RNA extraction.

2. Line 248 – change to Fig 2D

Thank you for alerting us to this oversight; we corrected it.

3. The authors discuss previous studies demonstrating only a minor contribution to chiA for invasion (lines 300-307). It should be worth mentioning in the text that one possible explanation could be redundancy, as the two chitinases investigated in the current work clearly present (Fig 1 & Fig 3), which might be overlooked in other studies.

Thank you for this suggestion. We added a statement discussing the possible redundant function of the two chitinases.
